# Failed Treatment of Classic Kaposi’s Sarcoma with Topical Timolol: Case Report and Review of the Literature

**DOI:** 10.7759/cureus.6272

**Published:** 2019-12-02

**Authors:** Rohit Gupta, Logan DeBord, Harry Dao

**Affiliations:** 1 Dermatology, Baylor College of Medicine, Houston, USA; 2 Dermatology, University of Colorado School of Medicine, Aurora, USA; 3 Dermatology, Loma Linda University, Loma Linda, USA

**Keywords:** kaposi's sarcoma, topical, timolol, classic kaposi sarcoma, case report

## Abstract

Classic Kaposi’s sarcoma (CKS) is an angioproliferative cutaneous neoplasm which currently lacks a well-defined treatment regimen. Because the disease is often localized, topical therapies offer therapeutic potential without the morbidity of systemic or surgical treatment. Timolol, a topical β-adrenergic receptor antagonist, has shown promise in the treatment of CKS in individual cases. Here we report a patient with classic Kaposi’s sarcoma who failed treatment with 0.5% topical timolol three times daily for 12 weeks. Topical timolol use has been previously reported in eight patients with CKS who all responded to treatment with no adverse effects. Our divergent experience from the literature implies that while topical timolol may be an effective and safe treatment alternative to traditional therapies for patients with CKS, further prospective studies are needed.

## Introduction

Kaposi’s sarcoma (KS) is an angioproliferative neoplasm of endothelial origin associated with human herpesvirus 8 (HHV-8) and human immunodeficiency virus (HIV) infection. Classic Kaposi’s sarcoma (CKS), one of four subtypes of KS, is characterized by dark-colored cutaneous lesions most frequently occurring on the distal extremities of the lower legs and feet in middle-aged to elderly males. Lesions develop slowly, and although CKS is rarely responsible for death, complications such as pain, ulceration, lymphedema, and bleeding are common [1]. Traditional management of CKS is usually individualized and targeted towards alleviating lymphedema, decreasing the size of lesions, and delaying or preventing disease progression with surgery, radiation, and/or chemotherapy. These treatments may cause significant adverse effects [2]. Localized topical therapies have unique potential for CKS, as the disease rarely involves lymph nodes, mucous membranes, or visceral organs [3]. Topical treatments for CKS could avoid subjecting patients to the cost of surgery or adverse effects of systemic chemotherapy or radiotherapy.

Timolol, a nonselective β-adrenergic receptor antagonist, is of particular interest in treating CKS due to its effectiveness in the treatment of infantile hemangiomas [4]. However, the use of timolol in CKS treatment has not been validated. Here we report failure of topical timolol in CKS. We also summarize and elucidate the current literature reporting the use of topical timolol in the treatment of CKS.

## Case presentation

**Figure 1:**
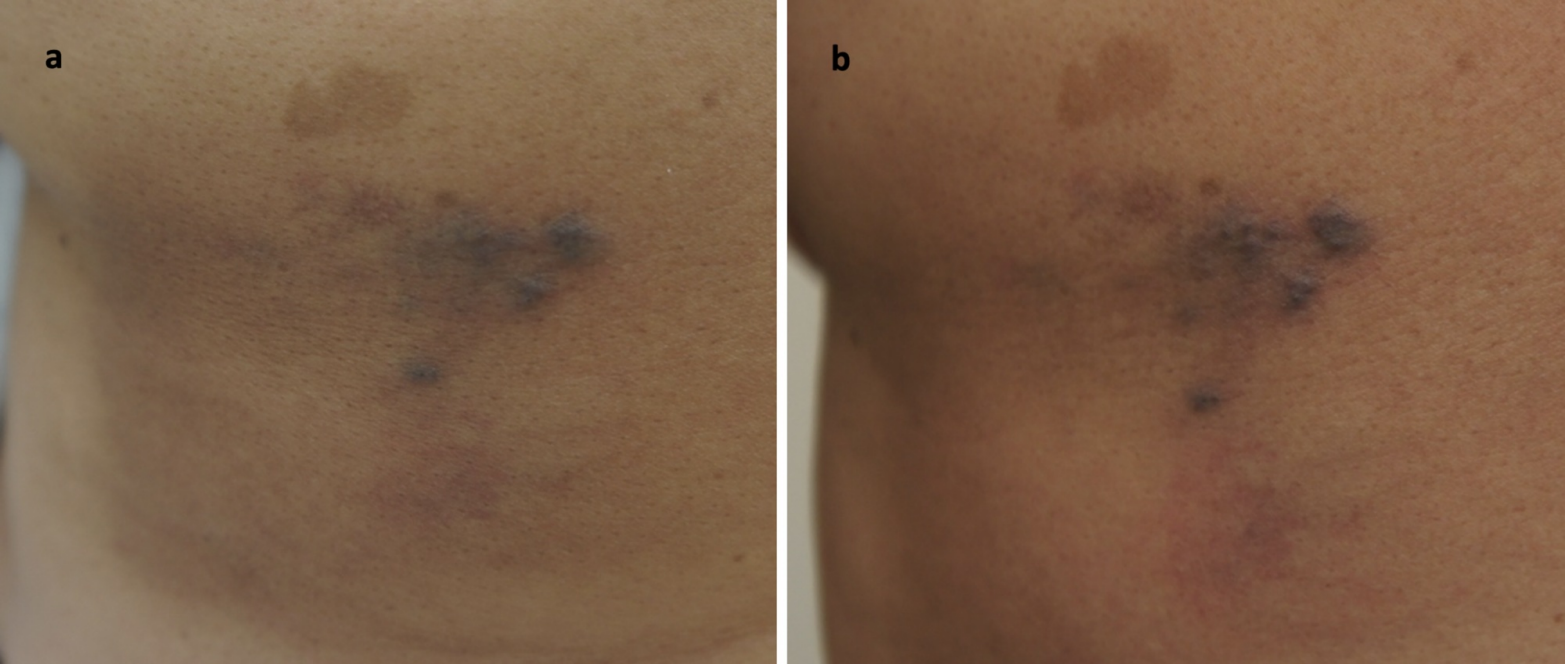
Classic Kaposi’s sarcoma, affecting the middle region of left mid-back. (a) Idiopathic, asymptomatic, well-delineated lesions. (b) No significant improvement at 12 weeks with 0.5% topical timolol solution applied three times per day.

A 55-year-old woman with a history of hypertension and colonic adenoma presented with a cluster of 6-10 moderately well-demarcated, dark purple papules and plaques ranging in size from 5 mm to 20 mm in the left mid-back region (Figure 1a). Histology revealed proliferation of spindle-like endothelial cells with slit-like vascular spaces and extravasation of red blood cells, consistent with CKS. Lesions were positive for HHV-8 following immunohistochemical staining, confirming diagnosis. The patient was HIV negative and was not taking any immunosuppressive drugs. With the diagnosis of CKS, the patient first received radiation therapy but without change in size or appearance of the lesions. She was then prescribed treatment with timolol ophthalmic gel-forming solution 0.5% twice per day, but she reported application of the solution to lesions three times per day. After 12 weeks of timolol application, although no adverse effects from treatment occurred, there was no improvement in size or appearance of the lesions (Figure 1b). Timolol treatment was stopped at this point, and the patient was started on local interferon alpha injections twice weekly.

## Discussion

To date, there have been eight cases published reporting the use of topical timolol to treat CKS (Table 1) [5-8]. Notably, the median age of reported cases was 74.5 years with a majority (75%) being male, consistent with the known demographics of CKS. Most patients (75%) presented with papules or plaques, although presentations also included discolored macules or bleeding nodules. All patients were HIV-negative and received topical timolol twice per day, with strengths varying between 0.1% and 0.5%. One patient was also receiving bisoprolol, an oral β-adrenergic receptor antagonist, to manage hypertension during timolol treatment; other medications of the remaining patients were not reported. All patients reported in the literature responded to treatment, with lesions rapidly remitting in 37.5% of cases following timolol treatment. Of the patients that experienced complete resolution, disease recurrence monitoring was not reported for three patients, but no others experienced recurrence at a mean follow-up of 12.3 months. Importantly, there were no adverse effects experienced from timolol treatment, with all but one case reporting data on adverse effects.

**Table 1:** Summary of case reports of CKS treated with topical timolol. CKS: Classic Kaposi’s sarcoma

Source	Sex, age	Location of disease	Clinical presentation	Timolol strength	Treatment duration	Treatment response	Follow-up, mo.
Gupta et al. (this report)	F, 55	Left mid-back	Purple papules	0.5%	12 weeks	No response	N/A
Abdelmaksoud et al., 2017 [5]	M, 52	Left leg	Plaque	0.1%	5 weeks	Complete resolution	10
Abdelmaksoud et al., 2017 [5]	F, 70	Left foot	Plaque & nodule	0.1%	5 weeks	Complete resolution	9
Abdelmaksoud et al., 2017 [5]	M, 65	Right leg	Plaque	0.1%	4 weeks	Complete resolution	6
Sainz-Gaspar et al., 2017 [6]	M, 71	Glans penis	Red-purple macules & papules	0.5%	24 weeks	Complete resolution	10
Alcántara-Reifs et al., 2016 [7]	M, 89	Medial toes bilaterally	Purple macules & nodules	0.5%	12 weeks	Complete resolution	5
Alcántara-Reifs et al., 2016 [7]	M, 83	Medial right ring finger	Bleeding, red nodule	0.5%	18 weeks	Resolution to residual macular appearance	4
Meseguer-Yebra et al., 2015 [8]	M, 78	Right foot	Erosive papule	0.5%	12 weeks	Complete resolution	22
Meseguer-Yebra et al., 2015 [8]	F, 94	Right leg	Papule	0.5%	12 weeks	Resolution to residual macular appearance	20

Our patient had a divergent outcome compared to previously reported cases of topical timolol for CKS, with the treatment failing to produce an effect after failed radiation therapy. This is in contrast to all other cases published in the literature, in which each patient achieved either partial or complete disease resolution. This may highlight the positive bias surrounding timolol treatment, as there may have been additional negative or equivocal results related to timolol use that were not published. While topical timolol may be an effective therapy for patients with CKS, it is important to consider this bias and plan for other treatment options if timolol treatment is attempted and fails.

Topical timolol has largely been shown to be effective in the treatment of infantile hemangiomas with a 96% response rate [4, 9]; in addition, evidence is emerging for its use in other conditions including pyogenic granulomas, recalcitrant wounds, chronic ulcers, and port-wine stains [10]. The mechanism of action of timolol is unclear but previous studies have shown that KS lesions are dependent on β-adrenergic signaling for the reactivation of HHV-8, resulting in proliferation of KS [11]. This finding supports a potential explanation for the reported effectiveness of timolol for CKS lesions on a molecular level.

Experience with timolol in other types of KS, including HIV-associated, endemic, and iatrogenic KS (usually resulting from immunosuppressive therapy), is limited. One case of timolol use in HIV-associated KS has been reported, where the use of 0.1% topical timolol twice daily led to remission at six weeks of treatment and no sign of recurrence at four-month follow-up [5]. Use of 0.5% topical timolol solution three times per day in iatrogenic KS following radiation therapy also improved ulceration and lymphadenopathy after 17 weeks in another case [12]. Neither of these cases reported adverse effects from treatment. While current evidence remains limited, these cases suggest that timolol may have potential to treat other forms of KS.

Timolol is one of a number of topical treatments emerging in CKS. Previous case reports have described clinical responses to 5% imiquimod [13], 0.5% rapamycin [14], and 0.1% alitretinoin [15]. However, with the exception of imiquimod, these have been reported as single case reports, with variable response [16].

## Conclusions

All cases in the current literature reporting the use of topical timolol treatment in CKS have demonstrated clinical response with no recurrence at follow-up for those that achieved complete resolution. However, this case report demonstrates that the treatment may not be effective in all patients. Together, this suggests that the use of topical timolol may be a safe and effective treatment option for individuals with localized CKS, but further controlled trials are warranted.
